# Reply to the Letter to the Editor Regarding “Description of Hip Deformities in 5-Year-Old Patients with Congenital Zika Virus Syndrome: A Cross-Sectional Study”

**DOI:** 10.1055/s-0045-1814118

**Published:** 2025-12-30

**Authors:** Herison Franklin Viana de Oliveira, Francisco Robson Queiroz Rego, Brauner de Souza Cavalcanti, Ítalo Rodrigues Bacellar, Thais Araújo Barbosa, Epitácio Leite Rolim Filho

**Affiliations:** 1 Departament of Surgery, Centro de Ciências Médicas, Universidade Federal de Pernambuco, Recife, PE, Brazil; 2 Departament of Orthopedics and Traumatology, Hospital Getúlio Vargas, Recife, PE, Brazil; 3 Departament of Orthopedics and Traumatology, Centro de Ciências Médicas, Universidade Federal de Pernambuco, Recife, PE, Brazil

We appreciate the pertinent observation regarding the risk of selection bias. We recognize that, as this is a retrospective cross-sectional study, this methodological limitation cannot be eliminated. However, we sought to reduce this bias as much as possible by including all children with congenital Zika virus syndrome who attended the outpatient clinic consecutively during the analyzed period, provided that they met the previously defined inclusion criteria. Thus, there was no arbitrary choice of cases, but rather the use of a representative sample of the population assisted in a reference center. We believe this strategy significantly reduced the risk of distortion in the results, even if it does not eliminate the limitations inherent in the retrospective design.

We agree with the observation that the sample size (62 patients) may limit more complex statistical analyses, especially when the data are stratified by gross motor function classification system (GMFCS) levels, which naturally reduces the number of individuals in each subgroup and, consequently, the accuracy and statistical power. We also recognize that the absence of confidence and control intervals for confounding variables, such as comorbidities or nutritional factors, is a limitation of the present study. However, we emphasize that its aim was to provide an initial description of hip structural changes in a specific population of children with congenital Zika virus syndrome, a topic still little explored in the literature. We emphasize that overcoming these limitations requires multicenter studies with larger numbers of participants and a prospective design, which will yield more robust analyses and better control of confounding variables.

We understand that the lack of methodological detail regarding covariate control may, in fact, have limited the interpretation of the results. However, we emphasize that, in our analysis, the variables Reimers index (RI), acetabular index (AI), and femoral neck-shaft angle (FNSA) were paired between patients who underwent tenotomy and those who did not, to detect statistical differences. 1 We recognize that this is a relevant point to improve in future versions and in subsequent studies with a larger number of cases.

We appreciate the considerations presented, especially regarding the issues raised to foster academic discussion. We recognize that such questions are extremely pertinent and enrich the reflection on the findings of our study. Although not directly explored in this analysis, we consider that these points represent relevant paths for future investigations and may support the development of new studies with more specific designs.

Suggestions regarding possible directions for future studies are valuable. We consider proposals for longitudinal designs to be extremely relevant, along with expanded and multicenter samples, and the incorporation of new technologies, such as three-dimensional (3D) modeling and artificial intelligence, to deepen understanding of the structural and functional changes of the hip in children with congenital Zika virus syndrome. Certainly, such approaches represent promising paths to generate more robust evidence and support individualized care strategies for this population.
